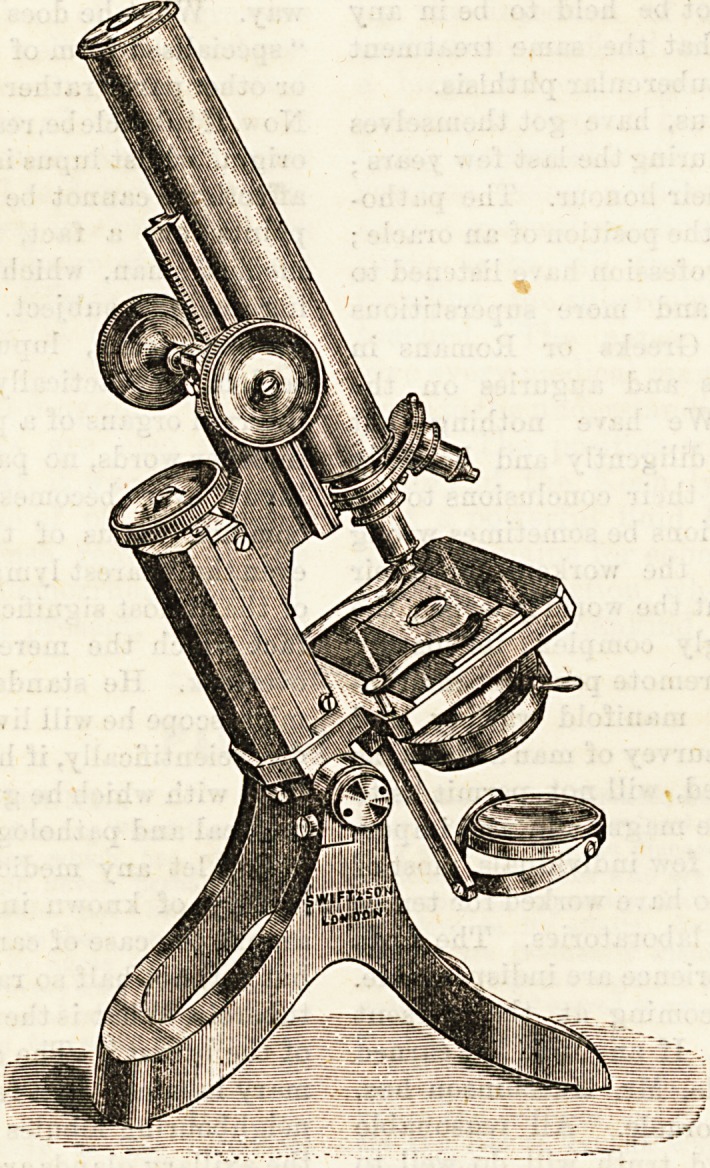# The Microscope in Medicine

**Published:** 1891-01-24

**Authors:** Frank J. Wethered


### The Microscope in Medicine.

By Frank J. Wethered, M.D.

### II.—THE USE OF THE MICROSCOPE.

Before passing to the subject matter proper of this article, a few more words are necessary as regards the description of the microscope. Most modern instruments have two adjustments, a coarse one, which serves to bring the lenses roughly into the focal position; this consists of either a telescopic joint, as in the Hartnach model, or of a rack and pinion movement, as in the English models. The fine adjustment is usually placed at the top of the body. Care should be taken to see that it revolves smoothly and uniformly. The lenses which are required vary with the uses to which it is proposed to put the instrument. Nearly every medical man of the present time wishes to examine specimens of sputa for tubercle bacilli, and in order to do this satisfactorily, an oil-immersion lens is necessary. The lens made by Leitz, which costs about £5 is thoroughly reliable; more expensive lenses are made by Zeiss. Some of the English makers, such as Swift and Baker, also make very good lenses. The other powers necessary are an inch, and a quarter, or, according to the continental systems, Nos. 3 and 7 of Hartnach, or A and D of Zeiss. In choosing these lenses it is always better for an inexperienced worker to seek the advice and help of one who has constantly used the microscope. The requisites of a good inch lens are that it should have a flat field—that is to say, all parts should be in focus at once, that it should possess a good definition, and be perfectly achromatic, so that when using it no circles of colour should appear round the edge.

A quarter-inch lens should have good penetration, so as to be able to examine rather thick sections, and at the same time have good revolving power, so as to be able to bring out clearly the most minute details of the specimen under examination.

It will be found to be a great convenience when working much with the microscope to have a "nose-piece" affixed, so that either a high or low power may be at once brought in connection with the tube. Not only is much time saved by this little piece of apparatus, but also it is far easier to examine any one spot which has been discovered by a low power with a high one, as the object glass is very apt to be shifted if one lens has to be taken off and another screwed on in its place; this is more particularly the case when working with the oil immersion lens.

Having thus described at length the instrument, of which the accompanying drawing shows an excellent model, we now pass to the consideration of how to handle the microscope.

This may seem somewhat superfluous, but a few hints to the inexperienced may save them not only various petty annoyances, but also probably the loss of valuable lenses. In removing the instrument from its box, it should be grasped by the column, not by its foot, as thereby much strain on its bearings is prevented. Any jarring of the adjustments soon puts them out of order. For this reason when in constant use it is preferable to keep it under a bell-jar rather than to be constantly lifting it out and into its case. Scrupulous cleanliness cannot be too strongly insisted upon. Each time the lenses are used they should be carefully wiped with a soft piece of chamois leather. This applies more particularly to the oil-immersion lens, for if the oil be allowed to remain on any length of time it is apt to become hard, and great trouble is experienced in removing it.

It not unfrequently happens when working with the high powers that these are accidentally forced through the cover glasses and so become covered with balsam; this is best removed by moistening the chamois leather with a little alcohol or xylol and rapidly passing it over the lens. This must be done very cautiously, as the lenses are "set" in balsam, and there is some danger of this being dissolved by the spirit, and if such be the case, the damage is extremely difficult to remedy.

Having settled the microscope on a firm table or stand (for any vibration is very disturbing) and inclined it at an angle to suit the convenience of the observer, the mirror is turned so as to reflect the light up the tube. If a low power is being used the flat side of the mirror should be employed, but if a high one, the concave side.

In conjunction with the mirrors, various diaphragms are employed to regulate the quantity of light passing to the specimen. In examining unstained objects, such as crystals and urinary deposits, the diaphragm with the smallest aperture is used with high powers. With stained specimens no regulation of the light is necessary. When using an oil-immersion lens the ordinary illuminator is not strong enough. What is known as a condenser (Abbe's being the best kind) is fixed below the stage in order to bring the rays of light to a focus. In the better class of microscopes this condenser is placed in the sub-stage, and by means of screws its focus can be accurately arranged. For temporary purposes its action can be dispensed with, either by lowering it or by placing a diaphragm between it and the mirror. The flat side of the mirror must always be employed with the condenser, which is intended to deal with parallel rays.

Two brass clips are arranged on the stage so as to fix the object glass. Some microscopists prefer to dispense with these altogether, but a very good plan, in order to steady the glass, is to fix it with the right hand clip, whilst the slide is moved about with the fingers of the left hand, the right being thus left free to turn the fine adjustment.

A few words will be found sufficient as regards the choice of eye-pieces. It is always best to use the lowest eye-piece, except when very great magnifying power is required; the reason for this being that a low eye-piece not only gives the best definition, but is also far less trying to the eye. Enlargement of the image is more advantageously secured by drawing out the tube of the microscope rather than by changing the ocular.

We now turn to the important point of the choice of the objective. The rule is—never use a higher power than is absolutely necessary for clearly bringing out the minutiæ of the object under examination. For ordinary medical work a quarter-inch is the highest that will be required, except for the examination of micro-organisms. When using this power, in order to avoid breaking the cover glass by injudicious use of the course adjustment, it is a good plan before applying the eye to the tube, to lower the lens until it just touches the cover-glass, and then to work it upwards by means of the fine adjustment whilst looking down the microscope. In using the oil immersion a drop of thick cedar oil is placed on the cover-glass, and the lens lowered until it comes into contact with the oil. The fine adjustment is then cautiously turned until the object comes into focus, and to prevent any accident it is best to slightly move the glass slide to and fro, as a moving point is more easily discernible than a stationary one. This completes the description and use of the microscope.

The only accessory piece of apparatus directly connected with the microscope that needs description here is the "camera lucida." The object of this instrument is to aid in the drawing of microscopical objects. A skilful draughtsman will scarcely need one, but to others it is a great assistance. There are a great many forms, but the simplest is known as "Beale's Neutral Tint Reflector," which consists of a small circular piece of tinted glass, which can be arranged at any angle to the eye piece. In using it the tube of the microscope is bent at right angles to the column, and a sheet of paper having been laid beneath the instrument, the left eye looks through the reflector and sees the false image reflected on the paper, whilst the right eye guides the movement of the pencil in tracing the outline. This is at first very difficult to do on account of the double image of the pencil which is formed, but a little practice will soon overcome this difficulty. It is a good plan merely to trace the chief features of the specimen by aid of the camera, and to fill in the details afterwards freehand, whilst observing the object in the usual way.

The size of an object is best measured by means of a micrometer; there are two kinds, "The Stage" and "Eye-piece." The former is used thus: If while one eye looks down the tube and the other is allowed to remain open, an image of the object will appear projected on the table at the side of the microscope, or on a piece of paper placed to receive it. Two points are then made, one at each end of the part to be measured. The preparation is then removed, and the stage micrometer fixed in its place. When this is focussed, fine parallel lines will be seen on it, equidistant from one another. These are generally one-hundredth or one-thousandth of an inch apart. An image of this is projected upon a sheet of paper in the same way as with the specimen. The distance between these two points being known, it is easy to compare them with the points previously made, and thus the size of the object is ascertained.

## Figures and Tables

**Figure f1:**